# Allodynia and Dysesthesia Associated With Semaglutide and Tirzepatide

**DOI:** 10.7759/cureus.94126

**Published:** 2025-10-08

**Authors:** Susan Ahern

**Affiliations:** 1 Endocrinology, Diabetes and Metabolism, David Geffen School of Medicine, University of California Los Angeles, Los Angeles, USA

**Keywords:** adverse drug event, allodynia, dysesthesia, glp1-gip receptor agonist, glp1 receptor agonist

## Abstract

Semaglutide and tirzepatide are two commonly prescribed glucagon-like peptide-1 (GLP-1) and GLP-1/glucose-dependent insulinotropic polypeptide (GLP-1/GIP) receptor agonists used for the treatment of type 2 diabetes and chronic weight management. Allodynia and dysesthesia have been previously reported as effects of semaglutide. This case report adds to the literature on dysesthesia from semaglutide and presents a novel report in a patient taking tirzepatide. This adverse drug reaction occurs in both patients treated for type 2 diabetes and chronic weight management, appears to be dose dependent, and resolves with discontinuation of the drug and may resolve spontaneously after several weeks.

## Introduction

This report details two cases of allodynia and dysesthesia from glucagon-like peptide-1 (GLP-1) and GLP-1/glucose-dependent insulinotropic polypeptide (GLP-1/GIP) receptor agonists. This side effect has only recently been reported after use of semaglutide in the literature since 2024 [1,2]. This is the first case report to describe this side effect associated with tirzepatide. In these cases, the medications were used for two different indications. In one case, tirzepatide was used in the form of Mounjaro® for type 2 diabetes. In the other case, semalgutide was used in the form of Wegovy® for the treatment of obesity. In both cases, the side effect occurred using the highest dose of each drug and had a spontaneous remission; however, in one case, the pain from tirzepatide reoccurred and was severe, leading to drug discontinuation and resolution of the allodynia.

## Case presentation

Case 1

A 56-year-old woman was started on liraglutide up to 3 mg subcutaneously daily for chronic weight management. She continued on liraglutide for six months with a gradual dose titration from 0.6 mg to 3 mg subcutaneously daily. She subsequently transitioned to semaglutide 2.4 mg weekly and began to complain of skin dysesthesia, describing her skin feeling like it had been sunburned, within a few weeks of starting semaglutide. The abnormal skin sensation persisted for approximately six weeks before resolving spontaneously. 

Case 2

A 75-year-old man with type 2 diabetes was taking oral semaglutide 14 mg by mouth daily. He was changed to subcutaneous tirzepatide and was increased from the lowest dose, 2.5 mg, monthly up to 15 mg. He reported allodynia after starting the highest, 15 mg weekly dose. He described the pain as a burning sensation all over his body. At baseline, he suffered from neuropathy and chronic back pain, already on pregabalin and duloxetine. Initially, his allodynia lasted two weeks and then resolved spontaneously; however, he experienced a second severe episode about six months later following treatment of a dental infection with antibiotics. The patient reported that the second episode was very severe and associated with odynophagia. There was no evidence of oral thrush. The tirzepatide was stopped, and the pain completely resolved. He was changed back to oral semaglutide 14 mg orally daily and tolerated this therapy well. He has subsequently taken up to 2 mg subcutaneously weekly of semaglutide and has not had any further allodynia and dysesthesia episodes.

## Discussion

GLP-1 and GLP-1/GIP receptor agonists are increasingly used for the treatment of type 2 diabetes and managing overweight and obesity [3]. Allodynia and dysesthesia have previously been reported as adverse reactions from semaglutide in two separate publications since 2024 [1,2]. In a review of 22 clinical trials and case reports, Tran et al. report that an oral 50 g dose of semaglutide caused dysesthesias, hyperesthesia, neuralgia, pain of skin, paresthesia, sensitive skin, and skin burning sensation. The incidence of each of these altered skin sensations was three to nine out of 334 patients. This paper also noted that one out of 407 patients reported dysesthesia from semaglutide 2.4 mg [1]. In 2025, Stark et al. shared a series of four patients who experienced allodynia from semaglutide 2.4 mg. Symptoms resolved in two patients who stopped the drug, and one patient continued the drug but experienced resolution after four months [2]. 

The data available from Tran and Stark, in addition to these reports, suggests that both oral and injectable doses of semaglutide in those treated for type 2 diabetes and chronic weight management may cause this side effect. Also, the effect may be dose-dependent, occurring at the highest doses of the drug. In some cases, the pain was reported to have resolved spontaneously after 6-16 weeks after the onset. In those who did not continue the medication, the pain also resolved after drug discontinuation [1,2].

This is the first published case report of allodynia and dysesthesia from tirzepatide. Despite numerous reports from patients on social media platforms, there are no formal case reports in the medical literature that document this phenomenon. In a published review of the data from the FDA adverse event reporting system, allodynia is listed among the top 50 adverse reactions reported while taking tirzepatide. This adverse reaction is not listed on the drug label [4].

Allodynia and dysesthesia are pain reported by a stimulus that would not generally cause pain. The etiology is not well understood, but it is thought to be an error in the long-term potentiation of sensory nerves. The symptom of allodynia is commonly associated with conditions such as fibromyalgia, trigeminal neuralgia, diabetic peripheral neuropathy, and migraine [5].

## Conclusions

This is the first published case report of tirzepatide-associated allodynia/dysesthesia. This side effect has been documented in semaglutide drug trials but is not currently listed on the drug label. These reactions appear to be dose-dependent and may resolve spontaneously over time and/or with discontinuation of the drug. Providers across many specialties, such as neurology and dermatology, as well as general practitioners, need to be aware of the potential side effects of allodynia and dysesthesia from semaglutide and tirzepatide, as these classes of medications are increasingly prescribed and new drugs in this class are in development. More research is needed to understand the mechanism, the incidence, and the risk factors for developing the adverse drug reaction, as well as the typical change over time with and without drug discontinuation. There is a need for greater awareness and for further research into this underrecognized adverse effect.
